# The role of family functioning: How the Big Five affect metacognitions about smartphone use

**DOI:** 10.3389/fpsyg.2022.991315

**Published:** 2022-10-06

**Authors:** Yuntian Xie, Qian Lei, Ruotong Xie, Yaping Yang

**Affiliations:** Department of Applied Psychology, Changsha Normal University, Changsha, China

**Keywords:** family functioning, metacognition, personality, smartphone use, the Big Five

## Abstract

The present study aimed to explore the relationship between the Big Five and metacognitions about smartphone use and the mediating role of family functioning. A cohort of 470 Chinese college students was selected as subjects based on the second edition of the Big Five Inventory-2, the Chinese version of the Metacognitions about Smartphone Use Questionnaire, and the general functioning subscale of Family Assessment Device. The results showed that only neuroticism was significantly and positively correlated with positive metacognition, while the correlation between other personality traits and positive metacognition was not statistically significant. Except for openness, the correlation between other personality traits and negative metacognition was statistically significant. In addition, conscientiousness, extraversion, and neuroticism were found to directly affect negative metacognitions about smartphone use and indirectly affect the negative metacognitions about smartphone use through family functioning. Findings provide insights into the design of interventions aimed at improving metacognitions about smartphone use and preventing smartphone addiction among college students.

## Introduction

With the continuous development and advances in society and technology, smartphones are now deeply rooted in the everyday lives of people all around the globe. The unlimited and increasing gratifications derived from smartphone usage have made people almost inseparable from their phones. For young college students, due to quasi-permanent access to the internet, smartphones are often used to fill in almost any gaps in their life (Harrison and Gilmore, 2012). Although smartphone use is accompanied by advantages such as sociability, entertainment, and information finding, it may also lead to adverse effects on physical and mental health due to dependence or addiction to mobile phones (Kayis et al., 2021; Alotaibi et al., 2022; Kwon et al., 2022).

### The Big Five and metacognitions about smartphone use

Metacognitions refer to information individuals hold about their own cognition and internal states, which affect their awareness, coping, and functioning (Wells and Matthews, 1996). According to the metacognitive model of psychopathology described by Wells, metacognition had an important role in all psychological disorders (Wells, 2013). The model suggests that specific metacognitive beliefs (also referred to as “metacognition”) are associated with the activation and maintenance of coping strategies (e.g., worry, rumination, threat monitoring, thought suppression, and coping behaviors) that lead to the persistence of psychological distress (Hamonniere and Varescon, 2018). In addition, Casale et al. (2021) suggested identifying metacognition associated with problematic technology use might have important clinical implications for treating maladaptive behavior patterns involving human-computer interaction.

Existing studies have found that metacognition is closely related to some types of technology addictions (e.g., IGD, Internet Gaming Disorder; PIU, Problematic Internet Use; PSU, Problematic Smartphone Use) (Casale et al., 2020; Hashemi et al., 2020; Aydın et al., 2021). Based on addictive behaviors, metacognitions could be divided into generic metacognitions about cognitive-affective experiences and specific metacognition about addictive behaviors, as both had a prominent impact on addictive behaviors (Hamonniere and Varescon, 2018). Further, the latter could be additionally divided into positive and negative metacognitions (Spada et al., 2015; Casale et al., 2020). Positive metacognitions relate to the effects of engaging in addictive behavior as a means of controlling and regulating cognition and affect (Spada and Wells, 2008; Spada et al., 2021). On the other hand, negative metacognitions refer to the dangers of one’s unmanageable thoughts related to addictive behaviors and thought-action fusion, leading to a lack of executive control over the engagement of addictive behaviors, and playing a crucial role in the perpetuation of addictive behavior (Spada et al., 2015). Thus, negative metacognitive beliefs are considered a primary factor contributing to the development and maintenance of psychological disorders (Huntley and Fisher, 2016) and appear to be more robustly correlated with these disorders than cognitions (Capobianco et al., 2020). Recently, there was a self-report to assess metacognitions about smartphone use, which demonstrated good psychometric properties in understanding the underlying mechanisms leading to problematic smartphone use (Casale et al., 2020). Shi et al. (2021) evaluated the psychometric properties of a Chinese version of the Metacognitions about Smartphone Use Questionnaire (MSUQ) and found that the Chinese MSUQ demonstrated promising reliability in assessing metacognitions about smartphone use in the Chinese-speaking population. Therefore, this instrument was used to measure metacognitions about smartphone use in the current study.

Among the factors related to metacognition, personality has received some attention from researchers (Cyrkot et al., 2021; Šafranj et al., 2021). As we know, personality has a complex underlying structure and may vary across populations and cultures (Smaldino et al., 2019). And the Big Five personality model has effectively summarized the important individual differences in people’s patterns of thinking, feeling and behaving, and answering questions regarding the structure of personality (Soto and John, 2017). So what does personality have to do with metacognition? One study pointed out that the Big Five was associated with metacognition, and openness was most closely linked to metacognition (Madison et al., 2021). Moreover, the personality trait of openness were found to be predictors of meta-comprehension judgments (metacognition) (Hacker et al., 2000). However, the study investigated metacognition in regard to the domain of reading rather than addiction. Additionally, Zaeske et al. (2022) revealed the influence of personality traits on flow metacognitions from the perspective of creativity. And some studies have explored metacognition in individuals with personality disorders (Cyrkot et al., 2021; Spada et al., 2021), metacognitive therapy has been applied to the study of personality disorders (Cheli et al., 2021; Simonsen et al., 2022).

Since the metacognition explored in this study is the metacognition of smartphone use, what is the relationship between personality and smartphones use? Some studies have found that personality traits could also be important factors contributing to problematic smartphone use or smartphone addiction (Demkow-Jania et al., 2021; Xiong et al., 2021). High neuroticism and low conscientiousness predicted problematic smartphone use well (Horwood and Anglim, 2021). A recent meta-analysis (Marengo et al., 2020) concluded that the Big Five personality traits could help understand individual differences in smartphone use disorder. However, throughout the available studies, the potential interplay between the Big Five and metacognitions in the field of addiction, especially metacognitions about smartphone use, is not well-developed. According to the Interaction of Person-Affect-Cognition-Execution model (I-PACE; Brand et al., 2016), person’s characteristics and cognitive responses can all contribute to the development of addictive behaviors. So what exactly is the relationship between the Big Five and metacognitions about smartphone use? Is the relationship between Big Five personality and positive metacognition the same as the relationship between Big Five personality and negative metacognition? Therefore, we proposed hypothesis 1.

**H1:** The Big Five may be significantly associated with metacognitions about smartphone use, including not only positive metacognitions, but also negative metacognitions.

### Mediation of family functioning

As a group, a family represents the cell of a society, which can be considered as the basic unit of social life. According to the McMaster family functioning model theory, the primary function of a family is to provide appropriate environmental conditions for each family member to develop and cope with their physical, psychological, social, and other needs (Epstein et al., 1983). It reflects on the structure of relationships, the flexibility of responses, the quality of family members’ interactions and the closeness between the family member (Beavers and Hampson, 2000). The type and quality of communication and the emotional ties related among family members are essential for the proper development of a member (Jimeno et al., 2022). Moreover, the personal characteristics of each family member, their interpersonal relationships, and how these are structured influence the development of younger member(s) (Carvalho et al., 2015). Poor family functioning could lead to the development of dysfunctional behaviors (Doba et al., 2014) and excessive smartphone use (Mangialavori et al., 2021). In addition, family factors such as punishment, reward, and nurturance can also influence members’ metacognition (Rani, 2020). Thus, the study speculated the existence of a strong relationship between family functioning and metacognition of smartphone use.

According to the person-situation interaction theory, an individual’s development is the result of constant interactions between the individual and his/her surrounding environment, within which distal factors of the ecosystem influence the individual’s development through the proximal environment (Magnusson and Stattin, 1998). Hou et al. (2018) stated that persistent and stable personality traits could be considered as distal factors, while family functioning could be considered as the proximal environment. While the personality traits of individuals, especially children, are influenced by the family (Nakao et al., 2000), each family member exhibits different personality traits that are influenced by the behavioral patterns and interpersonal relationships amongst the family members (Thielmann et al., 2020), which impact on the function of the family (Perveen et al., 2017). One study found that neuroticism and agreeableness could significantly predict family functioning in addicts, in which higher levels of neuroticism were associated with poorer family functioning and higher levels of agreeableness being were associated with better family functioning (Ahmadi et al., 2014). The present study seeks to fill the gap in the literature by examining the relationship between individual (personality) and environmental (family) factors as they influence metacognitions about smartphone use. Based on these observations, we proposed hypothesis 2.

**H2:** Family functioning may mediate the relationship between the Big Five and metacognitions about smartphone use.

**FIGURE 1:**
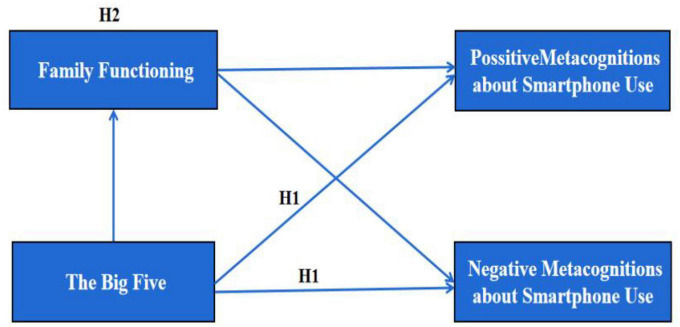
Conceptual model.

To summarize, this study aimed constructed a model of the relationships among the Big Five, metacognitions about smartphone use and family function (Figure 1).

## Materials and methods

### Participants

Convenience sampling was performed to select students from a university in Changsha, China. In all, 500 questionnaires were sent out. Excluding questionnaires with regular responses and questionnaires without responses on most topics, 470 valid questionnaires were returned back.

The effective recovery rate was 94.00%. There were students majoring in both liberal arts and science. Among the participants, there were 282 freshmen, 187 sophomores, and 1 person with missing grade information. Furthermore, there were 141 boys and 329 girls. The mean age of them was 18.47 (*SD* = 0.89).

### Measures

#### The Big Five

The second edition of the Big Five Inventory-2 (BFI-2) was compiled by Soto and John (2017) and translated by Zhang et al. (2021). The 60-item scale included the five dimensions of BFI-2, which comprised of openness, conscientiousness, extraversion, agreeableness, and neuroticism. A five-point scoring system was implemented whereby one referred to strongly disagreeing and five referred to strongly agreeing. Cronbach’s α of the study was 0.832 and the MacDonald’s ω was 0.847. The results of confirmatory factor analysis showed that the fit indexes of the model were relatively good, χ^2^/*df* = 2.818, TLI = 0.905, CFI = 0.899, RMSEA = 0.064. Additionally, AVE = 0.418, 0.429, 0.382, 0.403, 0.378, CR = 0.814, 0.862, 0.755, 0.781, 0.723.

According to Arpaci et al. (2022), convergent validity could be assumed for factors with lower than 0.50 AVE values if CR was higher than 0.60. Moreover, the square root values of the five factors AVE were greater than the value of correlation coefficient of this factor with other factors. It indicated that the discriminant validity of the scale was relatively good.

#### Metacognitions about smartphone use

The MSUQ compiled by Casale et al. (2020) and translated by Shi et al. (2021) was used in this study. The 24-items included the two dimensions of positive metacognitions (MSUQ-PM) and negative metacognitions (MSUQ-NM). A four-point scoring system was implemented whereby one referred to disagree and four referred to “strongly agree.” Cronbach’s α of the study was 0.893 and the MacDonald’s ω was 0.900. The results of confirmatory factor analysis showed that the fit indexes of the model were relatively good, χ^2^/*df* = 2.907, TLI = 0.901, CFI = 0.913, RMSEA = 0.065. Additionally, AVE = 0.438, 0.394, CR = 0.914, 0.862. Moreover, the square root values of both factors AVE were greater than the correlation coefficients. It indicated that the discriminant validity of the scale was relatively good.

### Family functioning

The general functional subscale of the Family Functioning Rating Scale compiled by Epstein et al. (1983) and translated by Wang et al. (1999) was also employed for this study. It consisted of 12 items. A four-point scoring system was implemented whereby one referred to very much like my home, and four referred to not at all like my home. Cronbach’s α of the study was 0.841 and the MacDonald’s ω was 0.846. The results of confirmatory factor analysis showed that the fit indexes of the model were relatively good, χ^2^/*df* = 3.248, TLI = 0.900, CFI = 0.926, RMSEA = 0.071. Additionally, AVE = 0.318, CR = 0.843.

### Procedure

The present study took advantage of the students’ concentrated self-study time to perform the test. The students all volunteered to participate in this study after understanding the purpose and requirements of the study. After the questionnaire was completed, it was collected on the spot. Overall, it took about 10 min to complete.

### Data analyses

First, we analyzed the reliability and validity of the measurement instruments used. SPSS 25.0 was used to calculate Cronbach’s α coefficient. Jamovi 2.2.5 was used to calculate MacDonald’s ω coefficient. Mplus 8.0 was used for confirmatory factor analysis. AVE and CR were calculated according to the corresponding equations. Next, we applied Mplus 8.0 to perform a common method biases test. Again, we applied SPSS 25.0 for descriptive and correlation analyses. Finally, based on this, we applied Mplus 8.0 to test the mediating effect of family function. In addition, ***, **, *, and ^Δ^ represent 0.1, 1, 5, and 10% significance levels, respectively.

## Results

### Common method biases test

Firstly, confirmatory factor analysis was conducted on a single factor model by combining the Big Five, metacognitions about smartphone use and family functioning into one factor. The results showed a poor-fitting effect of the single-factor model (χ^2^*/df* = 4.05, CFI = 0.18, TLI = 0.16, and RMSEA = 0.08). The Harman’s one-factor test was used to assess the common method biases, and the results showed that the first factor accounted for 10.66% of the variation, which was less than the 40% threshold; suggesting that there were no serious common method biases in this study.

### Preliminary statistics

Pearson’s product-moment correlation analysis (Table 1) showed that among the Big Five, only neuroticism was significantly and positively correlated with MSUQ-PM, while the correlations with other personality traits did not reach statistical significance. Except for openness, the correlation between MSUQ-NM and other personality traits was statistically significant. Among them, conscientiousness, extraversion, and agreeableness were negatively related to MSUQ-NM, while neuroticism was positively related to MSUQ-NM.

**TABLE 1:** Means, standard deviations, and correlations of the major variables.

Variable	*M* ± *SD*	1	2	3	4	5	6	7	8
1. BFI-O	3.13 ± 0.49	0.65							
2. BFI-C	3.21 ± 0.49	0.47***	0.65						
3. BFI-E	3.06 ± 0.55	0.36***	0.43***	0.62					
4. BFI-A	3.44 ± 0.55	0.43***	0.62***	0.38***	0.63				
5. BFI-N	2.92 ± 0.61	–0.01	−0.22***	−0.19***	−0.20***	0.61			
6. FF	3.06 ± 0.47	0.06	0.22***	0.28***	0.14**	−0.15**	0.56		
7. MSUQ-PM	2.35 ± 0.59	0.07	0.01	–0.01	0.09	0.11*	–0.02	0.66	
8. MSUQ-NM	1.87 ± 0.63	–0.05	−0.18***	−0.10*	−0.11*	0.28***	−0.17***	0.25***	0.63

BFI-O, openness; BFI-C, conscientiousness; BFI-E, extraversion; BFI-A, agreeableness; BFI-N, neuroticism; FF, family functioning; MSUQ-PM, positive metacognitions about smartphone use; MSUQ-NM, negative metacognitions about smartphone use. The diagonal numbers were the square root values of this factor AVE.

*p < 0.05, **p < 0.01, ***p < 0.001.

In addition, family functioning was negatively correlated with MSUQ-NM, and the correlation with MSUQ-PM was not statistically significant. Except for openness, the correlation between other traits and family functioning was statistically significant. Among them, conscientiousness, extraversion, and agreeableness were positively associated with family functioning, while neuroticism was negatively related to family functioning.

### Mediation test

Since only neuroticism was significantly correlated with MSUQ-PM among the Big Five traits, this study tested the mediating effect of family function between neuroticism and MSUQ-PM when constructing the mediation model. In addition, because the correlation between openness and MSUQ-NM was not significant, we did not test the mediating effect of family function on the relationship between openness and MSUQ-NM, but tested the mediating effect of family function on other personality traits and MSUQ-NM.

**FIGURE 2:**
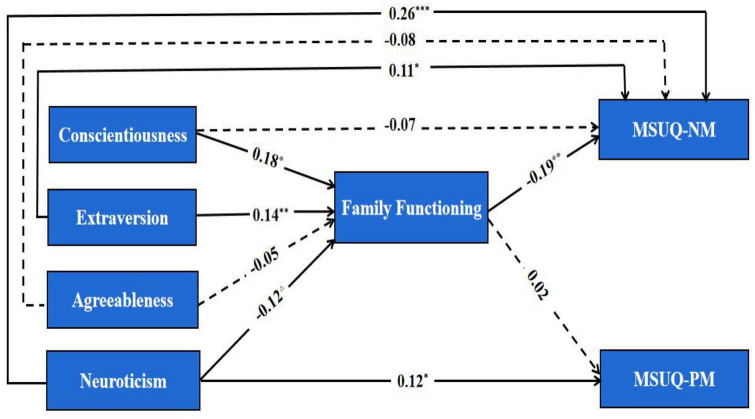
Mediation test about family functioning Δ*p* < 0.01, *p < 0.05, **p < 0.01, ***p < 0.001.

To investigate whether family functioning had a mediating effect on the relationship between personality and metacognitions about smartphone use, the Mplus software was used to assess the degree of fitting between the data and the hypothetical model (see Figure 2). The results showed that χ^2^/*df* = 1.21, CFI = 0.99, TLI = 0.98, and RMSEA = 0.02; indicating that the model had a good fitting degree.

As shown in Table 2, in the “Conscientiousness → FF → MSUQ-NM” path, the indirect effect values of the Boot CI did not include zero, suggesting that the mediation effect of family functioning was significant. In addition, the impact on MSUQ-NM became insignificant after the addition of family functioning (Figure 1), suggesting that family functioning could fully mediate the relationship between conscientiousness and MSUQ-NM. The mediating effect accounted for 16.67% of the total effect.

**TABLE 2:** Mediating effect of family functioning.

Mediation path	Indirect effect	SE	Boot CI	Relative mediation effect
1. Neuroticism → FF → MSUQ-PM	–0.01	0.01	[−0.02, 0.01]	9.09%
2. Conscientiousness → FF → MSUQ-NM	–0.03	0.02	[−0.08, −0.01]	16.67%
3. Extraversion → FF → MSUQ-NM	–0.03	0.01	[−0.05, −0.01]	30.00%
4. Agreeableness → FF → MSUQ-NM	0.01	0.01	[−0.01, 0.03]	9.09%
5. Neuroticism → FF → MSUQ-NM	0.02	0.01	[0.01, 0.05]	7.14%

Second, in the “Extraversion → FF → MSUQ-NM” path, the indirect effect values of Boot CI did not include zero, suggesting that the mediation effect of family functioning was significant. After adding family functioning as a mediator, the effect of extraversion on MSUQ-NM was still statistically significant; indicating that family functioning could partially mediate the relationship between extraversion and MSUQ-NM. The mediating effect accounted for 30.00% of the total effect.

In the “Agreeableness → FF → MSUQ-NM” path, the indirect effect values of the Boot CI included zero, suggesting that the mediation effect of family functioning was not significant, and that family functioning did not mediate the relationship between agreeableness and MSUQ-NM.

Lastly, in the “Neuroticism → FF → MSUQ-PM” path, the indirect effect values of the Boot CI included zero. However, in the “Neuroticism → FF → MSUQ-NM” path, the indirect effect values of Boot CI were not zero; suggesting that the mediating effect of family functioning was not significant in neuroticism and MSUQ-PM, but was significant in neuroticism and MSUQ-NM. After the addition of family functioning as a mediator, the effect of neuroticism on MSUQ-NM was still significant. This indicated that family functioning could partially mediate the relationship between neuroticism and MSUQ-NM. The mediating effect accounted for 7.14% of the total effect.

## Discussion

This study’s findings partially validated Hypothesis 1. As Ai et al. (2019) said, the Big Five was powerful in predicting smartphone-related activities in the Internet age, the results of this study showed that the Big Five was strongly associated with metacognitions about smartphone use. Specifically, we found that only neuroticism was positively correlated with MSUQ-PM, while the correlations with other personality traits were not statistically significant. In regards to MSUQ-NM, except for openness, the correlations with all personality traits were significantly correlated.

Several additional interesting observations were found between the Big Five and metacognitions about smartphone use. In the first place, a clear negative relationship between conscientiousness and negative metacognitions was observed. By definition, conscientiousness refers to being organized, decisive, disciplined, persistent, goal-oriented, and working hard to abide by rules and principles (Costa and McCrae, 1992). As a result, being conscientious about one’s activity could be beneficial to better manage addictive behaviors, including smartphone use, actively adjust to negative thoughts regarding uncontrollable thoughts and dangers of addictive behaviors, and would have a lesser likelihood of developing negative metacognitions. Second, we also observed a significant negative relationship between extraversion and negative metacognitions. Extraversion is one of the five core traits of the Big Five theory and is characterized by sociability, talkativeness, assertiveness, and excitability (Marino et al., 2018). Extraverts tend to view problems optimistically and respond decisively, and are therefore less likely to be controlled by negative thoughts. Third, a clear negative relationship between agreeableness and negative metacognitions was found. Agreeableness deals with motives for developing and maintaining positive prosocial relationships (Jensen-Campbell and Graziano, 2001). Individuals high in this trait often exhibit characteristics such as altruism, straightforwardness, and empathy, and thus, can also undermine the negative metacognition of smartphone use. Fourth, neuroticism was found to be closely related to both positive and negative metacognitions. Neuroticism refers to the loss of emotional adjustment and stability (McCrae and Costa, 1997), difficulties in balancing anxiety, hostility, impulsivity, depression, and other emotions. As a result, neuroticism has been associated with both high positive and negative metacognitions of smartphone use, and may lead to more pronounced smartphone addiction (Marengo et al., 2020; Horwood and Anglim, 2021). Last but not least, our findings also showed that openness and metacognitions about smartphone use were not closely related. Openness refers to individuals willing to try new things and is characterized by traits such as imagination, aesthetics, emotional richness, and difference seeking. Thus, considering that smartphone addictions usually refer to being physically stagnant for a long duration of time while all attention and experiences are drawn toward smartphone use, this could explain the lack of relation between openness and metacognitions about smartphone use.

In addition, this study found that family functioning had a significant mediating effect on the relationship between certain personality traits, i.e., conscientiousness, extraversion, and neuroticism, and MSUQ-NM. This observation partially validates Hypothesis 2. First, family functioning was shown to mediate the relationship between conscientiousness and MSUQ-NM. The present study found that conscientiousness positively affects family functioning. It was consistent with the existing research findings (Vollrath et al., 2010). Individuals with high conscientiousness can clearly master their roles in the family and cooperate with other family members, increase the members’ sense of belonging to the family, and reduce the occurrence of problematic behaviors (Hou et al., 2018). And good family functioning helps individuals to express their emotions (Ropi et al., 2021) and helps them to better regulate their feelings of lack of control. Consequently, they have lower levels of negative metacognition regarding smartphone use.

Furthermore, family functioning was found to mediate the relationship between extraversion and MSUQ-NM. This mediating effect accounted for a more significant proportion of the total effects, indicating a strong and important role of family functioning in linking extraversion to the MSUQ-NM. Further, we also found that extraversion and family functioning were significantly and positively correlated, which is consistent with the findings of a previous study (Pace et al., 2014). Enthusiastic and optimistic individuals often express their ideas and behave positively and proactively with other family members, promoting healthy family functioning and reducing the production of negative metacognition.

Last but not least, we observed that family functioning could also mediate the relationship between neuroticism and MSUQ-NM, suggesting that neuroticism could, directly and indirectly, influence MSUQ-NM through family functioning. Although the mediating effect of family functioning on this pathway did not make up a large proportion of the total effect, its status was still very important. Individuals who are emotionally unstable have difficulty communicating effectively and forming good interpersonal relationships, leading to poor family functioning. Studies have shown that poor family functioning occurs within families with high levels of conflict, disorganization and poor affective and behavioral control (Olson, 2011). Moreover, considering that poor family functioning can lead to an increasing level of negative metacognitions, if timely interventions are made to address the family functioning issues in this group, it would be likely that the negative metacognitions of its members could be significantly reduced. Further, this study did not find a mediating role for family functioning between personality traits and positive metacognition, nor a mediating role for agreeableness and MSUQ-NM. This may be related to both the connotations of positive metacognition and the characteristics of agreeableness. Future research is warranted to further validate this issue.

### Theoretical implications

Our findings have several theoretical implications. First, previous research has focused on the relationship between personality and metacognition in the learning domain (e.g., reading, Madison et al., 2021) and the application of metacognitive therapy to individuals with personality disorders, neglecting to explore the relationship between personality and metacognition in the smartphone addiction domain. To the best of our knowledge, this is the first study to explore the relationship between the Big Five and metacognitions about smartphone use. And it extended prior researches on metacognition in the smartphone addiction. The effect of personality traits on metacognitions about smartphone use also validated the I-PACE model’s view that personal factors could influence cognitive factors. We hope this research sparks further interest in advancing the literature on personality and metacognitions about smartphone use.

Moreover, we observed a mediating role of family functioning. It confirmed the person-situation interaction theory. As previously stated, personality was an individual factor and family functioning was an environmental factor. These two factors were not isolated from each other in influencing metacognitions about smartphone use, but were somewhat related. It suggested that Magnusson and Stattin’s (1998) perspective (i.e., that distal factors of the ecosystem influence individual development through the proximal environment) was correct. For the factors influencing metacognitions about smartphone use, we should see both the influence of personality and the influence of family functioning, as well as the relationship between personality and family functioning.

### Practical implications

Our findings have several practical implications. First, the relationship between personality traits and negative metacognitions about smartphone use should be given high priority in future studies and interventions that target metacognitive beliefs. In other words, different measures should be taken for individuals with different personality traits in order to guide individuals to adjust their negative metacognitions. As neuroticism has been found to be a risk factor for emotional distress and psychological disorders (Kayiş et al., 2016), the present study also found a closer association between neuroticism and both positive and negative metacognitions about smartphone use, thus requiring more attention to individuals with neuroticism. As stated by Wells (2019), we should pay attention to a sense of uncontrolled thinking and threat from cognition due to negative interpretations of thoughts and reduced perception controlling mental events.

Second, attention should be paid to the role of family functioning on metacognitions about smartphone use. Currently, there is a growing awareness of the importance of family functioning for individual growth. The subjects in this study were college students, most of whom were adults. For them, personality development is relatively stable, but family functioning can be improved. A meta-analysis including data from 24 countries (Olson et al., 2022) noted that smartphone addiction is increasing across the world, and China and Saudi Arabia had the highest rates of smartphone addiction. To change metacognitions about smartphone use and deal with the problem of smartphone addiction, we can start with family functioning, guide college students to communicate positively and effectively with other family members, and guide students’ parents to create a free and equal family environment.

### Limitations and directions for future research

There were some limitations to this present study that should be addressed. First, while we have identified the mediating role of family functions and emphasized the role of the family, the influence of the family on the lives of college students is, after all, relatively small. Future studies could select elementary or middle school students as subjects. The results so derived may be more informative. Second, smartphone addiction was not studied in this study, however, studies showed that personality and metacognition were all related to smartphone addiction. Future research could further reveal the relationship between these factors. Third, the data obtained from this study came from cross-sectional studies, and it was challenging to explain causal relationships. Although this study was based on person-situation interaction theory and considered personality as the independent variable and family functioning as the dependent variable, future studies could conduct cross-lagged analysis in order to further clarify the direction of the effect. In addition, the CR values of the scales used in this study were not high. The results of this study should therefore be interpreted with caution and replicated in future studies.

## Conclusion

Family functioning is a mediating factor between the Big Five and metacognitions about smartphone use. Conscientiousness, extraversion, and neuroticism can directly affect negative metacognitions about smartphone use and can indirectly affect negative metacognitions through family functioning.

## Data availability statement

The raw data supporting the conclusions of this article will be made available by the authors, without undue reservation.

## Author contributions

YX created a theoretical model, developed hypotheses, and revised the manuscript. QL collected the data and took part in drafting the manuscript. RX and YY proofread the manuscript. All authors contributed to the article and approved the final manuscript.
